# Acute changes in urinary metabolites: vinyasa yoga compared to cycle ergometer exercise

**DOI:** 10.3389/fspor.2025.1556989

**Published:** 2025-04-16

**Authors:** Colin E. S. Campbell, Carl J. Murphy, Zeinab Barati, Robert H. Coker

**Affiliations:** ^1^ Institute of Arctic Biology, University of Alaska Fairbanks, Fairbanks, AK, United States; ^2^ Montana Center for Work Physiology and Exercise Metabolism, University of Montana, Missoula, MT, United States

**Keywords:** urine, metabolism, exercise modality, nuclear magnetic resonance, unconventional exercise

## Abstract

**Introduction:**

Increased interest in unconventional exercise such as vinyasa yoga has outpaced our understanding of the physiological response to yoga exercise. The objective of the current study was to evaluate changes in urinary metabolites (i.e., alanine, phenylalanine, glycine, choline, taurine, creatinine, creatine, dimethylamine, citrate, pyruvate, acetate, and beta-hydroxybutyrate) elicited by vinyasa yoga compared to moderate intensity aerobic exercise in young healthy adults.

**Methods:**

Twelve participants, six women and six men, completed a vinyasa yoga exercise session (VY) and a moderate intensity cycle ergometer exercise session (ME) in a sequential fashion. The intensity of the ME was matched to heart rate and rating of perceived exertion elicited during the initial VY. Urine samples were collected at baseline and following the completion of each of VY and ME. Metabolite concentrations after each exercise were normalized to their baseline levels to obtain a relative exercise-induced change in concentration. We hypothesized that activation of large muscle groups in the lower extremities would foster greater ME-induced alterations in metabolites.

**Results:**

Exercise-induced changes in urinary concentrations of phenylalanine, creatinine, creatine, glycine, choline, taurine, dimethylamine, citrate, pyruvate, alanine, and beta-hydroxybutyrate were greater in ME compared to VY (*P* < 0.05). There was no difference between the exercise-induced changes in lactate between groups (*P* < 0.05).

**Discussion:**

The results of this study demonstrate that ME promotes more robust changes in urinary metabolites compared to VY. These differences may be due to a greater localized workload on the large muscle groups of the lower extremities during ME, and potentially highlight the distributed metabolic demand of VY.

## 1 Introduction

Vinyasa yoga exercise (VY) has become increasingly popular in westernized societies (1). Individuals who participate in yoga exercise gain improvements in flexibility, strength, endurance, and overall health (2). Whereas conventional aerobic exercise is typically performed using rhythmic sequences of light to moderate physical activity, VY is a relatively vigorous subtype of yoga that meets the criteria for moderate intensity aerobic activity using a distributed combination of strength and stamina postures (3). VY training programs utilize a combination of powerful movements, and muscular endurance that decreases chronic stress, improves heart rate variability and enhances quality of life (3). Intensity criteria for VY include 40%–59% of heart rate reserve, 64%–76% of heart rate_max_, 46%–63% of VO_2max_, and a rating of perceived exertion (RPE) of 12–13 or light to somewhat hard (4). Yoga exercise that meets these criteria promotes beneficial changes in oxygenated blood flow, autonomic balance, blood pressure, respiratory function, and orthostatic tolerance (5). Reports have also suggested that yoga may be one of the most effective means of metabolic disease prevention (6), but the protective mechanisms responsible are not well understood.

An overwhelming amount of research in the field of exercise physiology has well characterized the physiological adaptations for a wide range of exercise modalities (7). When the human genome was sequenced almost 25 years ago, the development of high-throughput omics techniques such as epigenomics, transcriptomics, proteomics, metabolomics and lipidomics provided scientists with new opportunities to evaluate metabolites that may have otherwise gone unnoticed or underappreciated (8, 9). Coupled with robust research designs and precision instrumentation, metabolomics technology can improve our understanding of physiological regulation and contribute to the development of new hypothesis driven investigations (8).

We know that lactate, pyruvate, and citrate are inextricably linked to exercise-induced changes in glycolysis and/or the tricarboxylic acid (TCA) cycle (10). Elevations in urinary alanine are linked by interrelated pathways and may reflect conversion from pyruvate to alanine via transamination (11). Exercise-induced alterations in urinary amino acids may also be elevated due to protein degradation (8). Alterations in creatine and creatinine seem variable, whereas dimethylamine implicated in the regulation of blood flow is modestly affected (12). Changes in beta-hydroxybutyrate reflect the augmentation of fatty acid metabolism (13). Moderate intensity exercise performed on a stationary cycle ergometer is a commonly used exercise modality in the scientific literature. Whereas vinyasa yoga exercise activates multiple muscle groups in a distributed fashion, moderate intensity exercise on a cycle ergometer relies more exclusively on skeletal muscle located in the lower extremities.

To our knowledge, there are no peer-reviewed studies in the literature that compare acute changes in metabolites during conventional exercise to VY, despite a growing interest in yoga in general. Therefore, there is a clear gap in knowledge in this regard. Based on studies that demonstrated greater acute changes in urinary metabolites during vigorous cycle exercise compared moderate cycle exercise (ME) (14), we hypothesized that the sustained engagement of large localized muscle groups would yield greater ME-induced alterations in metabolites that reflect greater local muscle metabolism in ME. The purpose of this study is to compare exercise-induced changes in urinary metabolites reflective of variations in metabolism, kidney function and/or blood flow during VY and ME.

## 2 Materials and methods

### 2.1 Study design and participants

The protocol, informed consent, medical history questionnaire, and a research personnel list were drafted and submitted to the University of Alaska Fairbanks (UAF) Institutional Review Board for review. Once approved, twelve research participants, including six males and six females were enrolled from the UAF campus and provided informed consent (Table 1). All participants completed a study questionnaire that included questions about their sex, age, dietary restrictions, exercise history (both present and past), tobacco and alcohol usage, medication usage, caffeine usage, and a comprehensive medical history questionnaire. Exclusion criteria included an existing history of substance abuse, cardiovascular, metabolic and/or cancer diseases, seizures, and any other conditions that might be exacerbated by fasting and/or exercise. All females were below the age of menopause, and no efforts were made to control the menstrual cycle.

**Table 1:** Clinical characteristics

Clinical characteristics	Yoga	Cycle	Yoga	Cycle	Yoga	Cycle
	Combined	Combined	Males	Males	Females	Females
Gender (M/F)	6/6	6/6	6	6	6	6
Age (years)	23.1 ± 7.1	23.2 ± 7.1	27.5 ± 4.5	27.5 ± 4.5	22.5 ± 2.4	22.5 ± 2.4
Body height (meters)	1.6 ± 0.4	1.6 ± 0.4	1.8 ± 0.1*	1.8 ± 0.1*	1.6 ± 0.1	1.6 ± 0.1
Body mass (kg)	63.2 ± 18.2	63.4 ± 18.1	71.9 ± 8.2*	72.0 ± 7.8*	62.5 ± 16.0	62.9 ± 16.0
Body fat mass (kg)	13.6 ± 8.3	13.6 ± 8.0	9.6 ± 2.1	9.9 ± 2.1	17.4 ± 11.1**	17.3 ± 10.7**
Body fat free mass (kg)	49.6 ± 15.8	49.8 ± 15.5	62.3 ± 6.4*	62.1 ± 6.0*	45.1 ± 5.2	45.6 ± 5.6
Total body water (kg)	36.3 ± 11.5	36.5 ± 11.4	45.6 ± 4.7*	45.4 ± 4.4*	33.0 ± 3.8	33.4 ± 4.1
Body mass index (kg/m^2^)	21.1 ± 5.6	21.2 ± 5.7	21.5 ± 1.4	21.6 ± 1.4	23.0 ± 4.9**	23.2 ± 4.9**
Body fat (%)	19.1 ± 8.9	19.1 ± 8.6	13.2 ± 1.9	13.6 ± 2.0	25.6 ± 9.1**	25.4 ± 8.5**
Basal metabolic rate (calories)	1,502 ± 429	1,504 ± 429	1,782 ± 153*	1,784 ± 146*	1,452 ± 166	1,456 ± 165

Males were significantly older than females in both trials (*P* < 0.05). Data are presented as Mean ± SD.

* Body height, body mass, body fat free mass, total body water, and basal metabolic rate was higher in males compared to females (*P* < 0.05).

** Body fat mass, body mass index and percent body fat were higher in females compared to males (*P* < 0.05).

Research participants were scheduled to attend two exercise sessions, the first session of which was 1 h of VY. The first session of VY established the steady state heart rate (i.e., ∼70% of heart rate_max_ and a RPE of 12–13) that would be replicated during the second session, which was 1 h of ME. This sequential approach standardized the relative intensity of the exercise based on heart rate and RPE. VY exercise pairs breath to movement; pairing moving the body into one yoga pose with the inhale, and then onto the next pose with the exhale. The common yoga poses include the following; down dog, updog, forward fold, halfway lift, warrior 1 & 2, extended side angle, child's pose, triangle, crescent lunge, runners lunge, pigeon pose. These poses were repeated 3x on each side of the body during the course of the VY session.

Participants were instructed to fast for 12 h and to drink at least 0.7 L of water 30 min prior to the exercise sessions to standardize their post-absorptive status and hydration levels, respectively. The fasting and water intake protocols were validated consistent with studies evaluating urinary metabolites (15).

Upon arrival for the VY and ME sessions, the height and weight of the participants was measured, and body composition was determined using a Tanita 300 bioelectrical impedance analyzer (Arlington Heights, IL). Participants were issued Soleus Flash heart rate monitors (Cedar Park, TX) and led through the VY session. RYT-200 (registered yoga instructor of a 200-hour certification course) which are accredited through yoga alliance led the VY session. Heart rate was documented at ten equally spaced intervals to ensure homogeneity during the VY session. One week following the VY session, the same participants returned for the ME session. Under direct supervision, participants were then instructed to complete the ME seesion at an intensity that matched their original recorded heart rate from the VY session. Using this approach, we standardized the exercise intensity of the VY session to the ME session. Participants were asked to consume their normal diet with limited variation between the two exercise sessions.

### 2.2 Sample analysis

Urine samples were collected prior to and immediately following cessation of the exercise sessions, placed in ice, and then frozen at −80°C until analysis. The risk of bacterial contamination was mitigated by using mid-stream urine collections (20). Once all data collection had been completed, urine samples were thawed and agitated in case any separation had occurred and then prepared according to previously published protocol (16). For NMR analysis, 400 μl of urine with 200 μl of pH 7.4 phosphate buffer were transferred into 5-mm NMR tubes (Wilmad Lab Glass, Buena, NJ). ^1^H–NMR spectra were acquired with a 600-MHz Bruker Avance-III system running TopSpin 3.2 software (Bruker Biospin, Fremont, CA) using a dual resonance high resolution SmartProbe with single axis Z-gradient. The water signal was suppressed using the SPR-w5-watergate sequence (17) with 64 scans, collecting 64k data points, with a 1 ms recycle delay. A standard, trimethylsilyl propionic-2,2,3,3-tetradeuteropropionic acid (TMSP in D_2_O) contained in a sealed tube and placed in the NMR tube was used for metabolite quantification of fully relaxed ^1^H–NMR spectra and as a ^1^H chemical shift reference (0.0 ppm). The ^1^H–NMR peaks for single metabolites were identified and referred to published chemical shift or a metabolite chemical shift library. All spectra were process according to previous methods (16). The NMR system is shimmed for every sample to ensure field homogeneity for improved spectral resolution, and pulse parameters are calibrated routinely to maintain proper flip angle in the experiments.

### 2.3 Data analysis

MetaboAnalyst 2.0 was used to analyze the data. Using the previous work of Pechlivanis et al., where the same research subjects completed two exercise bouts with either 10 or 60 s of rest, our calculation of statistical power using lactate as a variable was 1.00 with an alpha of significance of 0.001 (16). We normalized the metabolites concentrations after each exercise session relative to the pre-exercise levels. Shapiro–Wilk and Kolmogorov–Smirnov normality tests were used to test whether our dataset followed a normal (Gaussian) distribution. Since both tests rejected the null hypothesis (data follows a normal distribution), we used a non-parametric test, i.e., Wilcoxon Signed Ranks test. Mean differences were considered significant at (*P* < 0.05). This test is particularly useful for comparing potential differences between two related samples. The study was underpowered to evaluate sex-specific responses in metabolites in response to VY or ME.

## 3 Results

We recruited 12 participants for these studies (Table 1). Body height, body mass, body fat-free mass, total body water and basal metabolic rate was greater in males compared to females (*P* < 0.05). Body fat mass, body mass index and percent body fat was higher in females compared to males (*P* < 0.05) (Table 1). The average heart rate was 123 ± 19 beats/min during the VY, which was matched during the ME.

We identified 14 metabolites in our samples. These included phenylalanine, creatinine, creatine, creatinine, creatine, glycine, choline, taurine, dimethylamine, citrate, pyruvate, alanine, lactate, and beta-hydroxybutyrate. Except for lactate, exercise-induced changes in urinary concentrations of phenylalanine, creatinine, creatine, glycine, choline, taurine, dimethylamine, citrate, pyruvate, alanine, and beta-hydroxybutyrate were greater in ME compared to VY (*P* < 0.05) (Table 2).

**Table 2:** Urinary metabolites measured pre- and post-moderate exercise (ME) and pre- and post-vinyasa yoga (VY).

Metabolites	Phenylalanine (PPM)	Creatinine (PPM)	Creatine (PPM)	Choline (PPM)	Glycine (PPM)	Choline (PPM)	Taurine (PPM)	Dimethylamine (PPM)	Citrate (PPM)	Pyruvate (PPM)	Alanine (PPM)	Lactate (PPM)	BHB (PPM)
Pre-ME	55 ± 57	1,081 ± 1,177	204 ± 213	100 ± 115	57 ± 79	2,045 ± 2,163	93 ± 99	21 ± 24	106 ± 103	29 ± 30	16 ± 16	22 ± 24	25 ± 26
Post-ME	66 ± 79*	1,267 ± 1,509*	183 ± 175*	89 ± 95*	72 ± 86*	2,496 ± 2,906*	128 ± 140*	19 ± 19*	104 ± 94*	41 ± 47*	21 ± 24*	34 ± 41	26 ± 24*
Pre-VY	34 ± 26	652 ± 385	174 ± 125	56 ± 32	43 ± 37	1,292 ± 737	92 ± 66	13 ± 11	77 ± 53	20 ± 15	10 ± 6	14 ± 7	18 ± 10
Post-VY	33 ± 44	624 ± 884	132 ± 146	41 ± 44	44 ± 62	1,257 ± 1,730	113 ± 189	10 ± 14	68 ± 69	17 ± 21	11 ± 12	19 ± 21	17 ± 27

* Denotes significant exercise-induced difference between ME and VY.

## 4 Discussion

The purpose of this study was to examine changes in urinary metabolites in response to VY and ME matched for exercise intensity by heart rate. To our knowledge, this is the first study to describe intensity-matched, exercise-induced changes in urinary phenylalanine, creatinine, creatine, glycine, choline, taurine, dimethylamine, citrate, pyruvate, alanine, and beta-hydroxybutyrate that were greater with ME compared to VY. These changes were greater even though we matched the intensity of the exercise bouts based on heart rate. Fold changes in metabolites are directly linked to the work intensity and size of contracting skeletal muscles. The relative work rates of contracting large skeletal muscle groups in the lower extremities may be higher with ME than the work rates of more evenly distributed work rates of skeletal muscles during VY. As a result, these modality dependent work rates may have influenced more robust changes in metabolites associated with carbohydrate, fat, and protein metabolism in ME. The possiblity of sex-specifc responses to ME and VY were beyond the scope of this study, but could be affected by menstrual status or hormone fluctuations.

VY requires a greater overall distribution of mechanical movement compared to ME, utilizing more complex muscle activation, but potentially reducing the absolute metabolic demand on individual skeletal muscles. Pechlivanis et al., detailed the influence of rest intervals (10 vs. 60 s of rest) during sprint training on post-exercise urinary metabolites (16). These studies demonstrated that shorter rest intervals between sprinting bouts led to higher post-exercise urinary metabolites such as phenylalanine, glycine, alanine, dimethylamine, and beta-hydroxybutyrate. These authors posited that the shorter rest intervals reduced the time available for energy substrates to be replenished, therefore causing a greater metabolic disturbance (18). While both VY and ME were performed at a submaximal, steady state intensity, the interactive influence of localized bioenergetic demands and complex systemic pathways may have influenced the appearance of metabolites in the urine (19). In other words, ME demanded constant supply of energy substrate to a more localized muscle group as opposed to variable localized energy demands with VY, potentially elevating urinary metabolites derived from ME.

Given these results, one might question the use of urine as a biofluid for the evaluation of exercise-induced changes in metabolites between these two exercise modalities. Urine as a biological sample is a non-invasive strategy for clinical studies. However, the concentration of urine can be affected by hydration, diet and/or medications. Urinary lactate may not be as sensitive as blood lactate to acute changes in energy metabolism (20). By comparision, blood sampling provides more precise data but requires sterile instruments, the medical supplies to sterilize the blood draw site, a clinical environment in which to draw blood, and a medical professional that is credentialed to perform blood collection procedures. In addition, blood sampling presents a barrier to involvement for some research participants and there is a small risk of infection or inflammation at the site of blood sampling. While blood may show a more precise snapshot of metabolite levels, using it as a biofluid is more complicated.

An ancillary goal of this study was to combine least invasive approaches with H^1^–NMR based analytical approaches that could utilize urine as a bio-fluid. Metabolomics represents a mainstream analytical approach to obtain a greater understanding of metabolic processes. The use of a H^1^–NMR based metabolomics approach is a reasonable strategy to measure metabolites in human bio fluids that may be linked to metabolism, kidney function and/or blood flow (19), and can be applied to a wide range of health and exercise related studies in a culturally appropriate manner. Notably, all females were pre-menopausal and our study was not powered to examine sex-specific differences in urinary metabolites during VY and ME. This represents a signficiant limitation when interpreting these data. Future studies examining acute changes in plasma metabolities using H^1^-NMR may further detail changes in localized metabolism in the skeletal muscle across a continum of exercise modalities and intensities.

## 5 Conclusion

Significant differences in the exercise-induced changes in phenylalanine, creatinine, creatine, glycine, choline, taurine, dimethylamine, citrate, pyruvate, alanine, and beta-hydroxybutyrate between VY and ME in all metabolites but lactate were noted. These results suggest that localized metabolic rates may influence urinary metabolites and highlights the distributed metabolic demand on skeletal muscle bioenergetics during VY.

## Data Availability

The original contributions presented in the study are included in the article/Supplementary Material, further inquiries can be directed to the corresponding author.
